# Length-scale dependency of biomimetic hard-soft composites

**DOI:** 10.1038/s41598-018-30012-9

**Published:** 2018-08-13

**Authors:** M. J. Mirzaali, M. E. Edens, A. Herranz de la Nava, S. Janbaz, P. Vena, E. L. Doubrovski, A. A. Zadpoor

**Affiliations:** 1 Department of Biomechanical Engineering, Faculty of Mechanical, Maritime, and Materials Engineering, Delft University of Technology (TU Delft), Mekelweg 2, 2628 CD Delft, The Netherlands; 2 Department of Chemistry, Materials and Chemical Engineering Giulio Natta, Politecnico di Milano, Piazza Leonardo da Vinci, 32, 20133 Milano, Italy; 3 Faculty of Industrial Design Engineering (IDE), Delft University of Technology (TU Delft), Landbergstraat, 15, 2628 CE Delft, The Netherlands

## Abstract

Biomimetic composites are usually made by combining hard and soft phases using, for example, multi-material additive manufacturing (AM). Like other fabrication methods, AM techniques are limited by the resolution of the device, hence, setting a minimum length scale. The effects of this length scale on the performance of hard-soft composites are not well understood. Here, we studied how this length scale affects the fracture toughness behavior of single-edge notched specimens made using random, semi-random, and ordered arrangements of the hard and soft phases with five different ratios of hard to soft phases. Increase in the length scale (40 to 960 μm) was found to cause a four-fold drop in the fracture toughness. The effects of the length scale were also modulated by the arrangement and volumetric ratio of both phases. A decreased size of the crack tip plastic zone, a crack path going through the soft phase, and highly strained areas far from the crack tip were the main mechanisms explaining the drop of the fracture toughness with the length scale.

## Introduction

The building blocks of several natural composites with exceptionally good mechanical performance are two distinct phases: one hard and one soft. These phases are often arranged in a hierarchical multi-scale manner with functional gradients and considerable levels of heterogeneity^1–4^. In addition to the molecular structure of the hard and soft phases, these multi-scale arrangements play a key role in determining the mechanical performance of natural composites^3,5^ and enable them to exhibit simultaneously high levels of stiffness, strength, and toughness^4,6–8^; a combination that is otherwise hard to realize.

Different toughening mechanisms have been described for natural composites including ‘brick-and-mortar’ arrangement of hard and soft phases^9–11^, the presence of interconnected hard bridges^12,13^, the contact of nano-asperites at the nano-level^12,14^, and interaction of waviness of the constituents at the microscale^15–17^. These and other mechanisms cause energy dissipation at various length scales through crack blunting, crack branching, nucleation of micro-defects, and platelet pullout^14,15,18^. Synthetic composites mimicking this hard-soft combination of phases have been fabricated in the past through additive manufacturing^4,19–29^, 3D magnetic printing^26,30^, freeze casting and ice templating^13,31^, layer-by-layer deposition^32^, or foaming processes^33,34^.

The techniques used for fabrication of biomimetic composites are all similar in one aspect: the range of the lengths scales they could reliably and reproducibly achieve is limited^5^. The isolated effects of the achievable length scale on the stiffness, strength, and toughness of biomimetic hard-soft composites have not been studied before. Moreover, it is unclear how other design parameters such as the volumetric ratio of the hard and soft phases and their arrangements may influence the effects of the length-scale. Here, we present a detailed systematic study of both isolated and modulated effects of length scale on the mechanics of hard-soft composites.

## Materials and Methods

**Figure 1:**
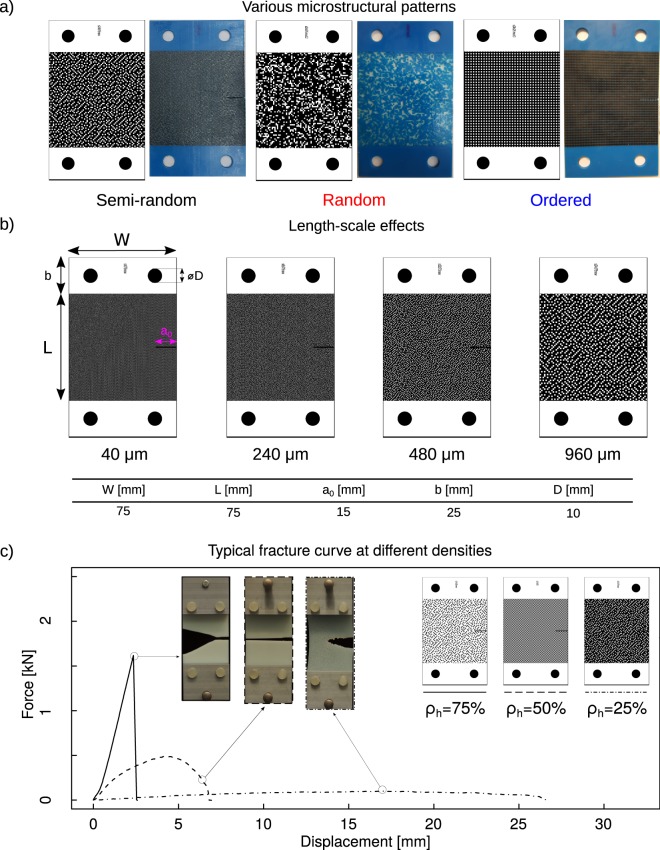
Single-edge notched tensile specimens with (**a**) different arrangements of hard and soft phases including semi-random, random, and ordered with *ρ*_*h*_ = 25%, (**b**) various length scales with *ρ*_*h*_ = 25%. The geometrical parameters of the specimens are given in the table. The binary images show the hard material bitmaps. (**c**) Typical force-displacement curves for semi-random specimens with different ratios of the hard to soft phase and a length scale of 960 μm.

We used a multi-material additive manufacturing technique (Objet350 Connex3 3D printer, Stratasys^®^ Ltd., USA) that inkjet-deposited droplets of photopolymer, followed by UV curing. The type of the material deposited through each droplet could be controlled at the minimal length scales of 40 μm × 80 μm × 30 μm^35^. The hard and soft phases of the composite were respectively printed using VeroCyan^TM^ (RGD841, shore hardness (D) 83–86) and Agilus30^TM^ Black (FLX985, shore hardness (A) 30–35), i.e., two commercially available materials for the 3D-printer. We used single-edge notched tensile specimens to evaluate the fracture toughness of the hard-soft composites (Fig. 1).

To define the composite structures, we used cuboids as the basic building blocks. Arranging the cuboids was done using three algorithms; random distribution, 3D error diffusion^36^, and 3D dispersed-pattern ordered dither to respectively create random, semi-random, and patterned hard-soft composites (Fig. 1a).

All these algorithms generate binary images that specify the distribution of the hard and soft phases (Fig. 1a and Figs 1 and 2 of the supplementary document). The final product was anisotropic in all three directions. We applied each algorithm to generate samples with five ratios of hard to soft cuboids (*ρ*_*h*_) of 0, 25, 50, 75, and 100%. For all combinations of algorithm type and *ρ*_*h*_ values, specimens were fabricated with four different length scales of 40, 240, 480 and 960 μm (Figs 1b and 3 of the supplementary document). The 40 μm length scale was made by dimensioning the cuboids at the smallest achievable length scale of the printer. For the three larger length scales, the cuboids were modeled with equal edge-length, meaning that the building blocks were cubes. This study design resulted in 38 experimental groups, which were tested in triplicates, resulting in a total of 114 specimens.

The initial crack spanned 20% of the specimen width and was perpendicular to the tensile loading direction. The specimens had a constant thickness of 3 mm. Both sides of the specimens were cleaned with ethanol after careful removal of the supporting material.

A gripping system and four pins were designed and additively manufactured using a fused deposition modeling (FDM) 3D printer (Ultimaker 2+, Geldermalsen, The Netherlands) from polylactic acid (PLA) filaments (MakerPoint PLA 750 gr Natural). The part of the specimens that was fixed in the gripping system was made from the same hard material as the one used in the composite. The specimens were attached to the gripping system via pins. In the case of fully hard specimens, aluminum fixtures were used.

Fracture toughness tests were performed under displacement control using an LLOYD instrument (LR5K) mechanical testing machine with a 5 kN loadcell and a stroke rate of 2 mm/min. The time, force, and displacement were recorded at a sampling rate of 20 Hz. The crack initiation, crack path, and crack propagation paths were analyzed using digital microscopy (Keyence^®^ vhx-5000) at different magnifications (20–200x) with a zoom lens (VH-Z20T).

Full-field strain measurements were performed during the mechanical tests of the specimens with the largest and smallest length scales using the digital image correlation (DIC) technique. A speckle pattern was created by randomly spraying black dots on a white background that was applied to one side of the specimens. A commercial DIC system including two digital cameras (4 MP with CMOS chip) and the associated software (Vic-3D 1, Correlated Solutions, SC, USA) was used to determine the strain distribution.

The normal stress, *σ*, was defined as the ratio of force, *F*, to the effective cross-sectional area, ${A}_{0}=t\times (w-{a}_{0})$, of the specimens. The strain, *ε*, was defined as the ratio of the displacement, *u*, to the initial free length between the grippers, ${L}_{0}$. The stiffness, *E*, was calculated using a moving regression algorithm with a box width of 0.2% strain to measure the stiffest part of the loading. The fracture stress, ${\sigma }_{f}$, was defined as the maximum stress. The fracture toughness was calculated from the numerical integration of the area under the stress-strain curve until the end of the test (final fracture).

## Results and Discussion

Three distinct types of fracture were observed in the specimens depending on the hard to soft ratio, *ρ*_*h*_ (Fig. 1c). The specimens with *ρ*_*h*_ values of 75% showed brittle fracture where the stress linearly increased until fracture (Fig. 1c). The stress-strain curves of these specimens were similar to the monolithicly hard ones, albeit with a lower level of fracture stresses (Fig. 1c). The majority of the specimens with a *ρ*_*h*_ value of 75% (31 out of 36 specimens) showed a crack bridging usually located at the center of the specimen. The specimens with *ρ*_*h*_ values of 50% and 25% showed ductile fracture and a non-linear stress-strain behavior (Fig. 1c).

**Figure 2:**
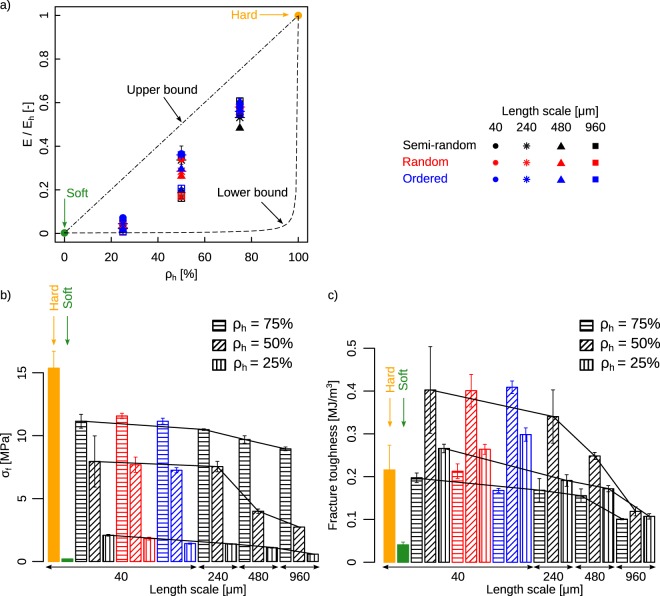
Comparison of the fracture properties of the specimens with different arrangement of the hard and soft phases, length scale, features, printing resolutions, and *ρ*_*h*_ values. (**a**) The stiffness of the composite specimens and comparison with the upper and lower bounds obtained from the rule of mixtures. Bar plots of the fracture stress (**b**) and fracture toughness (**c**).

The monolithicly hard and soft specimens defined the upper and lower boundaries of the elastic modulus and the fracture stress (Fig. 2a). The fracture stress and stiffness of the composite structures cannot, therefore, exceed those of monolithicly hard specimens.

**Figure 3:**
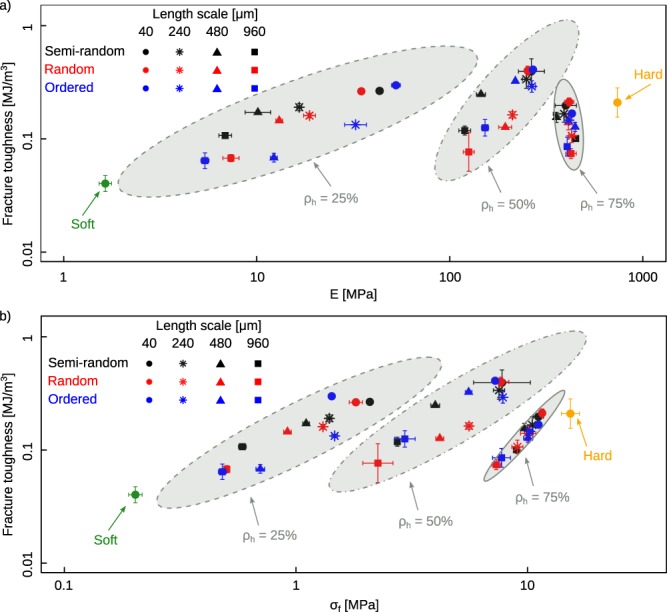
The log-log plot of the fracture toughness vs. stiffness (**a**) and fracture stress (**b**). For each *ρ*_*h*_, an ellipse was fitted to the data points to mark the 95% confidence intervals.

The stiffness values of the monolithic hard and soft specimens were respectively 739.86 ± 26.71 and 1.65 ± 0.13 MPa (mean ± SD). A higher volumetric ratio of the soft phase resulted in lower stiffness values regardless of the type of the arrangement of both phases (Fig. 2a). This reduction in the stiffness is, however, not necessarily proportional to the amount of the added soft phases (Fig. 2a). The range within which the stiffness values varied was broader for the specimens with equal amounts of hard and soft phases (i.e. *ρ*_*h*_ = 50%) (Fig. 3a). The length scale and arrangment of the hard and soft phases affected the stiffness values the most when a significant amount of the soft phase was present in the composites (Fig. 3a and Table 1 of the supplementary document).

To put this in perspective, increasing the length scale from 40 μm to 960 μm had almost no effect on the stiffness of the specimens with *ρ*_*h*_ = 75% while causing a two-fold drop in the stiffness when *ρ*_*h*_ = 50% (Fig. 3a and Table 1 of the supplementary document). The same trend was observed across all types of arrangements of the hard and soft phases (Fig. 3a and Table 1 of the supplementary document). The drop was even higher (i.e., seven folds) for the specimens with *ρ*_*h*_ = 25% (Fig. 3a and Table 1 of the supplementary document). This clearly shows how the ratio of the soft phase modulates the effects of the length scale on the stiffness of the hard-soft composites.

The fracture stresses were respectively 15.36 ± 1.34 MPa and 0.2 ± 0.01 MPa for the monolithicly hard and soft specimens (Fig. 2b and Table 1 of the supplementary document). Increasing the ratio of the soft phase reduced the fracture strength (Fig. 2b), as the soft compartments act as weak spots in the structure where the crack could more easily propagate through. Increasing the length scale from 40 μm to 960 increases the size of such weak spots and makes it easier for the crack to find them. That is why the fracture stress drops significantly, as the length scale increases (Figs 2a and 3b). The arrangement of the hard and soft phases did not substantially affect the fracture stress of the composites (Fig. 2b).

The specimens with equal amounts of hard and soft phases (i.e. *ρ*_*h*_ = 50%) exhibited the highest levels of fracture toughness. For the smallest length scale, i.e. 40 μm, the fracture toughness of the composites with *ρ*_*h*_ = 50% exceeded that of the hard phase (Fig. 2c) regardless of the arrangment type of the hard and soft phases. The difference between the fracture toughness of the composites with different *ρ*_*h*_ values decreased with the length scale (Fig. 2c). The type of the arrangement of the hard and soft phases generally influenced the fracture toughness more, when the ratio of the hard phase was smaller (Fig. 2c, Table 2 of the supplementary document).

As is clear from the Ashby plots (Fig. 3), the fracture toughness values found here are within the range of those reported in reference^20^ that designed nacre-inspired composites based on the mineral bridging found in those materials. The mineral bridges act as crack deflectors. Here, however, we did not design the arrangement of the hard and phases a priori. Instead, we applied different algorithms that generate a structure of the hard and soft phases, given the desired local ratio of the two phases. Figure 3 also shows that designing architectural arrangements of the microstructure of the composite material gives more degrees of freedom (wider ranges of stiffness values) when the density of the hard phase is low or intermediate. Indeed, for *ρ*_*h*_ = 25% and 50%, a wider range of the stiffness values could be obtained compared to that could be achieved at 75%.

**Figure 4:**
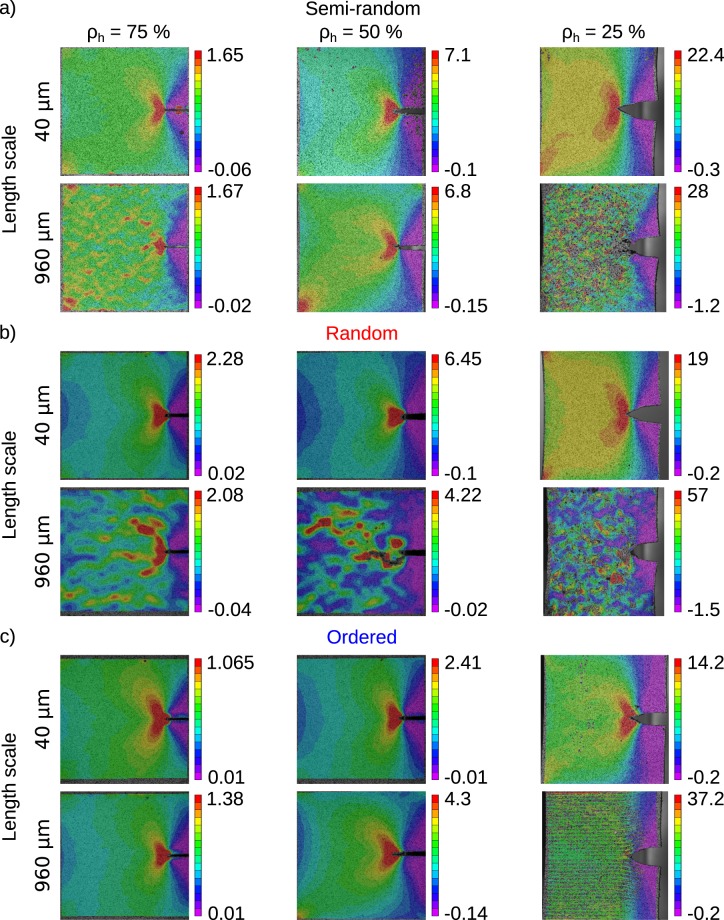
The DIC images of the specimens with semi-random (**a**), random (**b**), and ordered (**c**) arrangements of the hard and soft phases and extreme values of the length scale at three volumetric ratios of hard materials. DIC images correspond to the maximum stress values. All strain values are presented in %.

**Figure 5:**
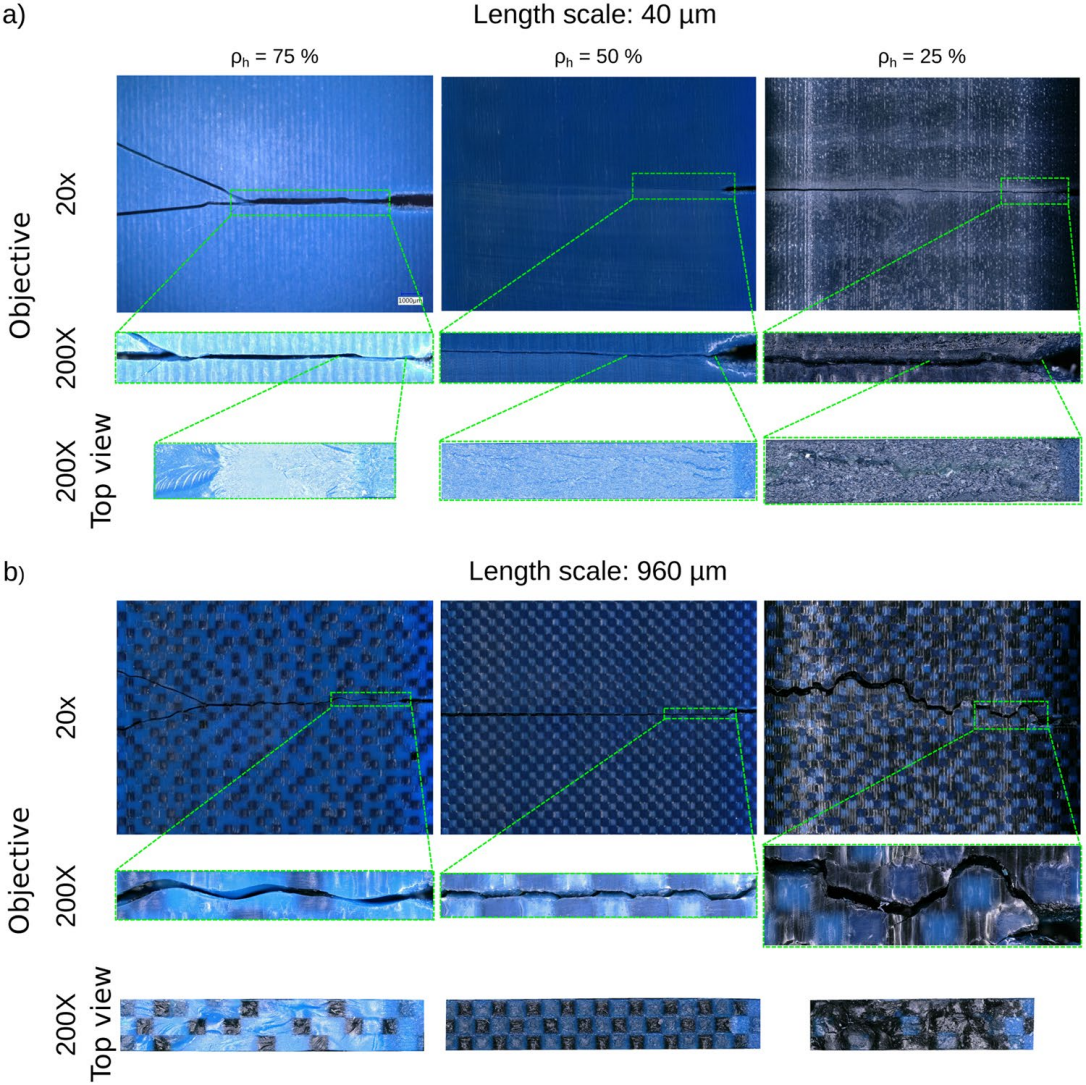
Microscopic images of the crack path for the semi-random specimens with length scales of 40 μm (**a**) and 960 μm (**b**). The images have equal scale bars and are captured at magnifications 20X and 200X (first two rows). The images appearing in the third row are the fracture surfaces (top view) photographed at a magnification of 200X.

Full-field strain measurements performed with DIC (Figs 4 and 4 of the supplementary document) and microscopic analysis of the crack path (Figs 5, 1 and 2 of the supplementary document) explained the mechanisms behind the above-mentioned observation regarding the effects of the printing length-scale and how other parameters such as the ratio of the soft (hard) phase and arrangement of hard and soft phases modulate those effects.

The root cause of fracture, similar to most other materials, is stress concentration around initiated cracks that lead to crack opening and propagation and ultimately failure. The crack initiation phase of this process is usually governed by the stochastic processes that define the incidental occurrence of weak spots and/or local overloading. In addition to these usual mechanisms, the fracture of such composites depends on the amount of soft material in the composite structure. The higher the proportion of the soft phase, the higher its influence on the mechanical behavior of the composite (including fracture behavior). For instance, the fracture behavior of composites with *ρ*_*h*_ = 75% was similar to the monolithically hard ones, with the soft phase not contributing much to the fracture properties. When the hard phase was decreased to 25%, the fracture behavior was similar to the monolithic soft specimens. For these slightly softer composites, softer building blocks mainly contribute to the total fracture of the composites.

Clear crack blunting was observed for specimens with high amount of the soft phase, i.e. *ρ*_*h*_ = 25% (Fig. 4b). In contrast, there was no sign of crack blunting when *ρ*_*h*_ = 75%. The size of the butterfly zone usually associated with crack tip plasticity decreased with the length scale as well as the amount of the hard phase (Fig. 4a). Indeed, no clear crack tip plastic zone could be oberved for many of the specimens with large length scales. Since the plastic zones dissipate energy, a larger size of the plastic zone results in higher values of the fracture thoughness. That is one of the reasons why the fracture toughness decreases as the length scale increases.

Moreover, a small length scale (e.g., 40 μm) resulted in the high strain areas being limited to the vicinity of the crack tip, while areas with very high values of strain were observed far from the crack tip in specimens whose length scale was large (e.g., 960 μm) (Fig. 4a). Highly strained areas far from the crack tip suggests that the large size of the length scale has enabled the stress to find a pathway through the weakest links of the composite (i.e., the soft phase) and reach further distances. These pathways could later facilitate crack growth and lower the energy cost of fracture. That explains why the fracture toughness decreases with the length scale. This is further confirmed with the type of the crack paths found in specimens with different sizes of the length scale. The microscopic images of the crack paths (200x) clearly showed sharp straight lines when the length scale was small (e.g., 40 μm), while wavy crack paths were found when the length scale was large (e.g., 960 μm) (Fig. 5). Further analysis of the microscopic images showed that, when the length scale was large, the crack propagated either through the soft phase or at the interface of the hard and soft phases (Figs 3 and 5 of the supplementary document). The hard phase in the composite works as a crack deflector and higher stress is required to break those hard phases as compared to the soft ones. A smaller length scale forces the crack to propagate through the hard phases, thereby increasing the fracture toughness. Smaller length scales also results in higher levels of energy required at the molecular scale to break the polymeric chains at the interfaces^37^.

Indeed, for all hard phase concentrations (75%, 50%, and 25%), the composites with finer features (the smallest length scales) exhibited higher toughness (approximately one order of magnitude higher than that of the larger microstructures). This might be explained by the confining mechanism acted by the hard phase on the soft domain which features a smaller characteristic length (smaller scale). As the characteristic size of the hard inclusions decreases (smaller scale), the matrix ligament thickness decreases being it proportional to the particle size^38^. This enhances the mechanical properties of the matrix.

The fact that the optimum ratio of the hard and soft phases for maximizing the fracture toughness is 50% is an important observation of the current study. The highest toughness found at 50% is then the result of an increased capability to withstand strain in the soft phase, in comparison to the 75% case, with a limited strength reduction. This result is also consistent with the observation that natural materials like lamellar cortical bone which is a soft/hard composite material are characterized by approximatively 50% of the hard component (the hydroxyapatite) and 50% by the soft component (the collagen matrix)^39^. Given that multiple competing mechanisms drive the fracture behavior of the specimens, one expects that there should be an optimum value for the ratio of each phase. For example, a specific range of the ratio of the soft to hard phase contributes towards higher fracture toughness through enhanced crack deflection. Very high ratios of the soft phase will, however, make it easier for the crack to propagate through the soft phase, thereby decreasing the fracture toughness. The exact ratio of the soft and hard phases to optimize specific properties (e.g., fracture toughness) cannot, however, be predicted at presence, as extensive theoretical studies are required to quantitively study the role of each competing mechanism.

## Conclusion

In summary, we found the length scale to affect the fracture behavior of hard-soft biomimetic composites both independently from other design parameters and in modulation with those. Both fracture stress and fracture toughness decreased between 2–4 folds, as the length scale increased from 40 μm to 960 μm. A decreased size of the crack tip plastic zone, a crack path going primarily through the soft phase (or through the interface of the hard and soft phases), and highly strained areas far from the crack tip appear to be the main mechanisms causing the lower values of the fracture energy when the length scale is large. In addition to better understanding the performance of bio-inspired composite structures, the new insights gained through this study could be applied in the slicer of multi-material printers, where algorithms need to convert the continuous material definitions into structures that are composed of discrete phases. The information obtained through the studies similar to the current one could be used by the slicer to generate the structure of the deposited cuboids such that the printed part meets the design criteria.

## Electronic supplementary material

Supplementary Material
