# Supplementary material for: Length-scale dependency of biomimetic hard-soft composites
Source: Sci Rep. 2018 Aug 13;8:12052. doi: 10.1038/s41598-018-30012-9 (PMC6089912; doi:10.1038/s41598-018-30012-9)
Supplement: Supplementary file 1 — Supplementary Material [file 41598_2018_30012_MOESM1_ESM.pdf]

Supplementary document to

Length-scale dependency of biomimetic hard-soft composites

M. J. Mirzaali[a,*], M. Edens[a], A. Herranz de la Nava[a], S. Janbaz[a], P. Vena[b], E. L. Doubrovski[c], A. A. Zadpoor[a]

[a] *Department of Biomechanical Engineering, Faculty of Mechanical, Maritime, and Materials Engineering, Delft University of Technology (TU Delft), Mekelweg 2, 2628 CD, Delft, The Netherlands*

[b] *Department of Chemistry, Materials and Chemical Engineering Giulio Natta, Politecnico di Milano, Piazza Leonardo da Vinci, 32, 20133 Milano, Italy*

[c] *Faculty of Industrial Design Engineering (IDE), Delft University of Technology (TU Delft), Landbergstraat, 15 2628 CE, Delft, The Netherlands*

[*] Corresponding author. Tel.: +31-15-2783133
E-mail address: M.J.MirzaaliMazandarani@tudelft.nl ; mirzaali.mohammad@gmail.com.

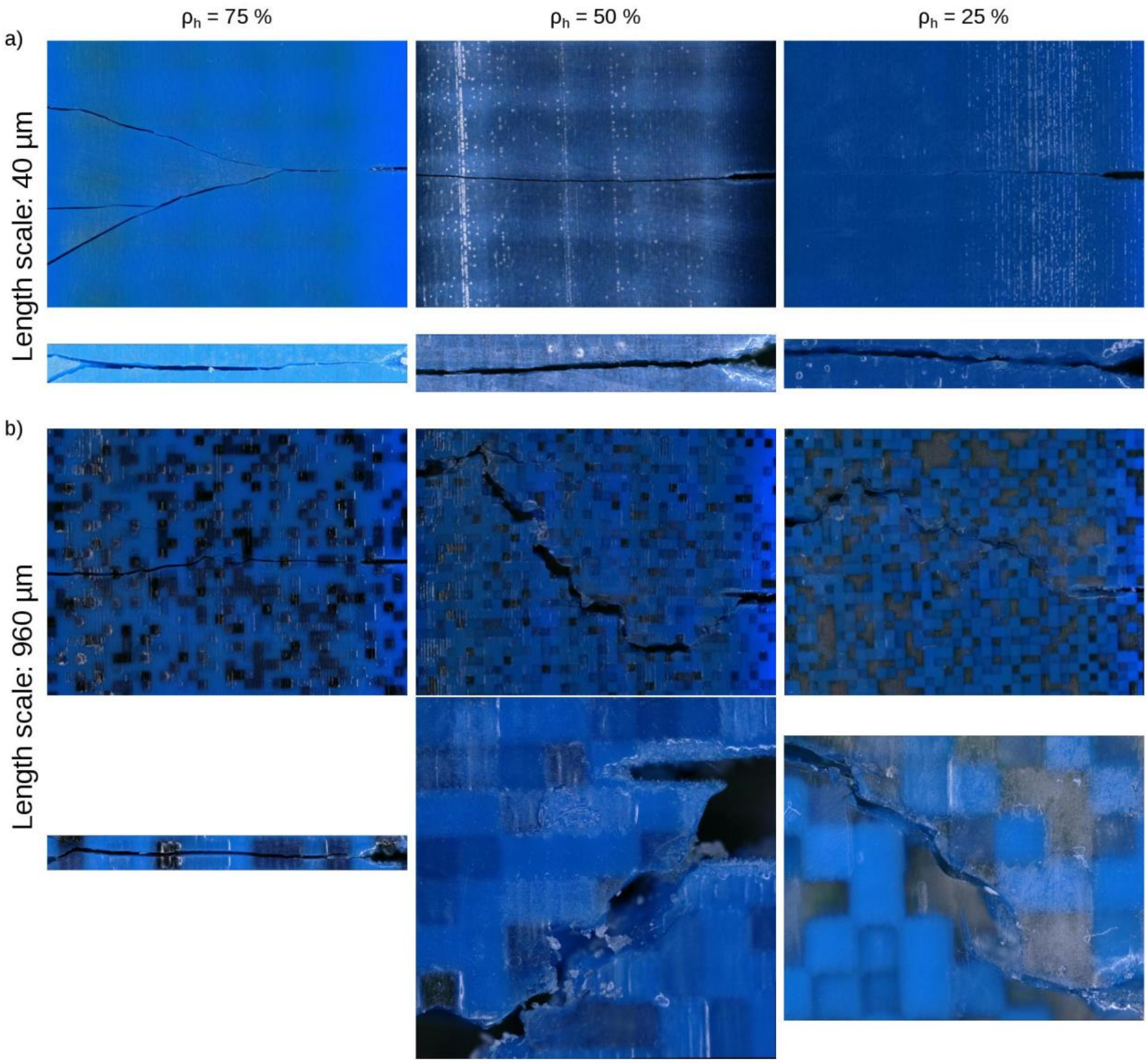

Figure 1. Microscopic images of the specimens with random microstructural arrangements at the printing resolution of a) 40 μm, and b) 960 μm. The images have similar scale bars and are captured at the magnification of both 20X and 200X for each specimen.

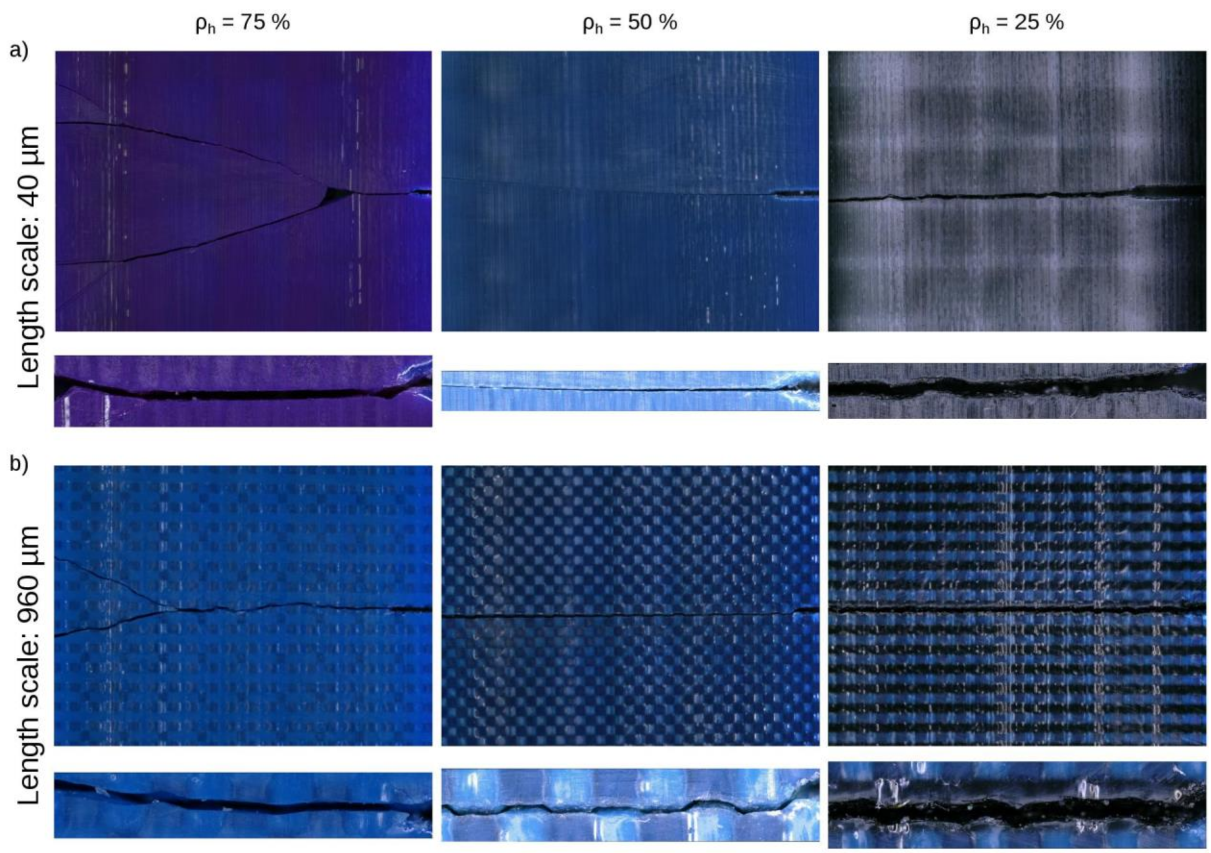

Figure 2. Microscopic images of the specimens with ordered microstructural arrangements at the printing resolution of a) 40 μm, and b) 960 μm. The images have similar scale bars and are captured at the magnifications of both 20X and 200X for each specimen.

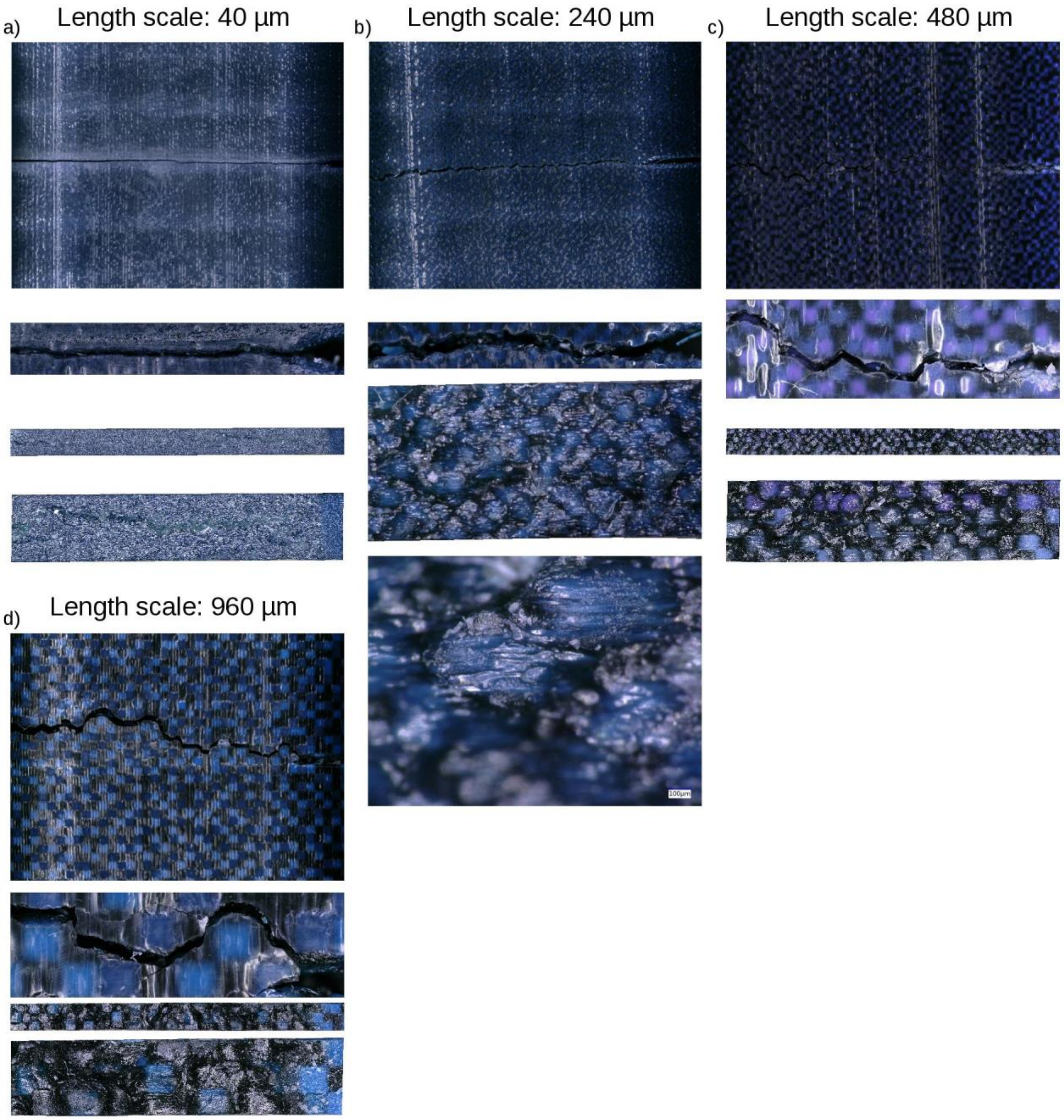

Figure 3. Microscopic images of the specimens with semi-random microstructural arrangements at the printing resolution of a) 40 µm, b) 240 µm, c) 480 µm, and d) 960 µm. The images have the same scale bars and are captured at magnifications 20X and 200X for the first two rows. Images in the third and fourth rows are fracture surfaces captured at magnification 50x and 200X.

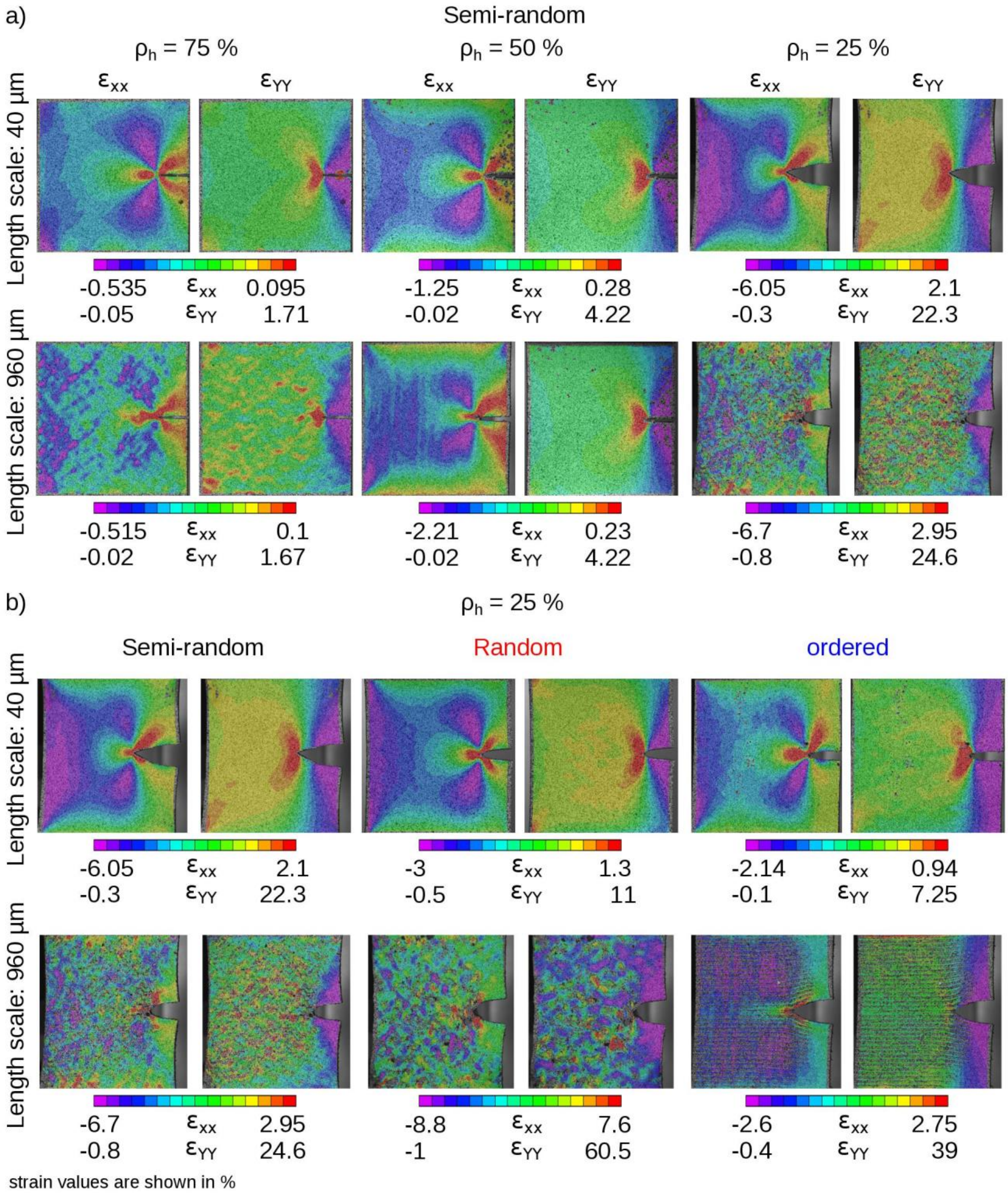

Figure 4. a) DIC images of the specimens with the semi-random arrangement of the hard and soft phases and extreme values of the length scale. b) DIC images of specimens (ρ_h =25%) with different types of arrangement of the hard and soft phase and extreme values of the length scale. DIC images correspond to the maximum stress values. All strain

values are presented in %. X is the horizontal and Y is the longitudinal direction with respect to the crack.

Table 1. The mean ± standard deviation of the fracture properties of 3D printed composites with various densities and microstructural arrangements.

Table 1

	Monolithic		Type of pattern											
	Hard	Soft	Semi- Random											
Voxel size [µm]	40	40	40	240	480	960	40	240	480	960	40	240	480	960
ρh [%]	0	100	75				50				25			
E [MPa]	739.86 ± 26.71	1.65 ± 0.13	401.48 ± 50.78	393.39 ± 52.68	357.41 ± 11.02	449.33 ± 3.38	226.92 ± 40.49	249.28 ± 11.69	145.36 ± 7.82	119.86 ± 8.54	43..34 ± 8.54	16.60 ± 0.49	10.22 ± 1.48	6.88 ± 0.53
σf [MPa]	15.36 ± 1.34	0.20 ± 0.01	11.15 ± 0.55	10.48 ± 0.06	9.69 ± 0.30	8.95 ± 0.15	7.95 ± 2.04	7.54 ± 0.41	4.00 ± 0.18	2.74 ± 0.02	2.09 ± 0.02	1.39 ± 0.03	1.11 ± 0.04	0.59 ± 0.02
Fracture toughness [MJ/m3]	0.22 ± 0.06	0.06 ± 0.01	0.20 ± 0.01	0.17 ± 0.03	0.16 ± 0.02	0.10 ± 0.00	0.40 ± 0.10	0.34 ± 0.06	0.25 ± 0.01	0.12 ± 0.01	0.27 ± 0.01	0.19 ± 0.01	0.17 ± 0.01	0.12 ± 0.01
			Random											
Voxel size [µm]			40	240	480	960	40	240	480	960	40	240	480	960
ρh [%]			75				50				25			
E [MPa]			415.90 ± 22.85	428.98 ± 15.00	411.64 ± 10.57	425.44 ± 27.09	253.31 ± 8.29	211.24 ± 1.32	194.13 ± 14.46	125.71 ± 8.00	34.84 ± 0.92	18.79 ± 1.35	13.08 ± 0.54	7.38 ± 0.70
σf [MPa]			11.57 ± 0.21	9.05 ± 0.49	10.08 ± 0.75	7.34 ± 0.32	7.69 ± 0.61	5.59 ± 0.17	4.18 ± 0.17	2.28 ± 0.35	1.82 ± 0.12	1.31 ± 0.02	0.92 ± 0.03	0.50 ± 0.01
Fracture toughness [MJ/m3]			0.21 ± 0.02	0.11 ± 0.01	0.14 ± 0.02	0.07 ± 0.01	0.40 ± 0.04	0.16 ± 0.01	0.13 ± 0.01	0.08 ± 0.03	0.26 ± 0.01	0.16 ± 0.01	0.15 ± 0.00	0.07 ± 0.01
			Ordered											
Voxel size [µm]			40	240	480	960	40	240	480	960	40	240	480	960
ρh [%]			75				50				25			
E [MPa]			429.80 ± 6.04	414.30 ± 14.33	446.23 ± 5.10	406.20 ± 15.41	269.90 ± 8.56	266.37 ± 11.64	218.49 ± 4.03	152.55 ± 6.56	52.64 ± 2.76	32.70 ± 4.09	12.27 ± 0.51	5.40 ± 0.25

σf [MPa]	11.15 ± 0.24	10.22 ± 0.35	10.07 ± 0.47	7.74 ± 0.70	7.25 ± 0.20	7.82 ± 0.10	5.57 ± 0.18	2.96 ± 0.30	1.43 ± 0.05	1.47 ± 0.04	0.70 ± 0.03	0.48 ± 0.02
Fracture toughness [MJ/m3]	0.17 ± 0.00	0.15 ± 0.01	0.13 ± 0.01	0.09 ± 0.02	0.41 ± 0.01	0.29 ± 0.03	0.32 ± 0.01	0.13 ± 0.02	0.30 ± 0.02	0.13 ± 0.00	0.07 ± 0.01	0.06 ± 0.00

Table 2. The relative ratio of the fracture properties with respect to the corresponding specimens with 50% hard ratio.

Table 2

	Type of pattern											
	Semi- Random											
Voxel size [µm]	40	240	480	960	40	240	480	960	40	240	480	960
ρh [%]	75				50				25			
E [MPa]	1.77	1.58	2.46	3.75	1.00	1.00	1.00	1.00	0.19	0.07	0.07	0.06
σf [MPa]	1.40	1.39	2.42	3.27	1.00	1.00	1.00	1.00	0.26	0.18	0.28	0.22
Fracture toughness [MJ/m3]	0.50	0.50	0.64	0.83	1.00	1.00	1.00	1.00	0.68	0.56	0.68	1.00
	Random											
Voxel size [µm]	40	240	480	960	40	240	480	960	40	240	480	960
ρh [%]	75				50				25			
E [MPa]	1.64	2.03	2.12	3.38	1.00	1.00	1.00	1.00	0.14	0.09	0.07	0.06
σf [MPa]	1.50	1.62	2.41	3.22	1.00	1.00	1.00	1.00	0.24	0.23	0.22	0.22
Fracture toughness [MJ/m3]	0.53	0.69	1.08	0.88	1.00	1.00	1.00	1.00	0.65	1.00	1.15	0.88
	Ordered											
Voxel size [µm]	40	240	480	960	40	240	480	960	40	240	480	960
ρh [%]	75				50				25			
E [MPa]	1.59	1.56	2.04	2.66	1.00	1.00	1.00	1.00	0.20	0.12	0.06	0.04
σf [MPa]	1.54	1.31	1.81	2.61	1.00	1.00	1.00	1.00	0.20	0.19	0.13	0.16
Fracture toughness [MJ/m3]	0.41	0.52	0.41	0.69	1.00	1.00	1.00	1.00	0.73	0.45	0.22	0.46
